# The Multifaceted Menace of *Fusarium* as a Plant, Animal, and Human Pathogen

**DOI:** 10.3390/biology15060453

**Published:** 2026-03-10

**Authors:** Kavindya Abeysinghe, Asanka Madhushan, Ahmed Mahmoud Ismail, Evgeny Ilyukhin, Sajeewa S. N. Maharachchikumbura

**Affiliations:** 1 School of Life Science and Technology, Center for Informational Biology, University of Electronic Science and Technology of China, Chengdu 611731, China or 202524140107@std.uestc.edu.cn (K.A.); or 202414140110@std.uestc.edu.cn (A.M.); 2 Pests and Plant Diseases Unit, College of Agricultural and Food Sciences, King Faisal University, P.O. Box 420, Al-Ahsa 31982, Saudi Arabia; amismail@kfu.edu.sa; 3 Independent Researcher, Swift Current, SK S9H 4E6, Canada; evgeny.ilyukhin@gmail.com

**Keywords:** zoonotic fungi, genomic plasticity, accessory chromosome, mycotoxin, one health, climate change

## Abstract

*Fusarium* is a diverse genus of filamentous fungi that has long been recognized for its importance in plant disease and food security. Beyond its agricultural impact, a growing number of studies now show that *Fusarium* species can also act as opportunistic pathogens in animals and humans. This review synthesizes current knowledge on *Fusarium* biology by integrating perspectives from plant pathology, veterinary science, and medical mycology. We examine how shared virulence mechanisms, environmental reservoirs, and genomic plasticity—including accessory chromosomes and horizontal gene transfer—facilitate adaptation across plant, animal, and human hosts. We also consider the role of environmental change in shaping the distribution and pathogenic potential of this genus. By bringing together evidence that is often scattered across disciplines, this review emphasizes the need to move beyond host-specific views and highlights *Fusarium* as a useful model for understanding fungal adaptability and cross-kingdom pathogenicity within a One Health framework.

## Simple Summary

*Fusarium* is widely known for causing devastating crop diseases and contaminating food with harmful toxins. However, it is increasingly recognized as a pathogen that can also infect animals and humans. This ability to cross host boundaries raises important concerns for agriculture, public health, and environmental management. This review explores how *Fusarium* survives in diverse environments and why it can infect such different hosts. By considering plant, animal, and human infections together, we highlight the need for coordinated strategies to manage this globally significant fungal threat under a One Health framework.

## 1. Introduction

The genus *Fusarium* comprises a diverse group of filamentous fungi that colonize an unprecedented range of ecological niches across the plant and animal kingdoms. It belongs to the phylum Ascomycota and currently comprises more than 430 species and about 3500 isolates and hybrids that are non-classified [1]. *Fusarium* creates colonies with velvety to cottony surfaces that are white, pink, red, purple, salmon, or gray in hue [2]. They are frequently found in nature within soil and decomposing organic material, where they can exist in both pathogenic and nonpathogenic relationships with plants, animals, and other organisms, with some species acting as saprobes and others living as endophytes within plants [1,3,4]. *Fusarium* species are responsible for a wide range of economically significant agricultural diseases that impact cereals, legumes, fruits, and ornamentals globally, including wilts, rots, and blight [5,6]. The resulting yield losses, coupled with mycotoxin contamination of crops, have profound implications for global food security and national and international trade [7,8,9]. Once primarily regarded as notorious plant pathogens causing extensive agricultural losses [4,10], *Fusarium* species have now been recognized as significant opportunistic pathogens of animals and humans [11]. This dual role as both environmental saprophytes and cross-kingdom pathogens underscores their remarkable adaptability and complex pathogenic potential.

Some *Fusarium* species have emerged as opportunistic pathogens in animals and humans, especially in immunocompromised individuals. *Fusarium fujikuroi, F. oxysporum*, *F. solani* (now *Neocosmospora solani*) species complexes have been particularly associated with disease manifestations beyond plant hosts [12,13,14,15]. These infections range from superficial keratitis and onychomycosis to invasive, life-threatening fusarioses [16,17,18,19].

Their remarkable genomic flexibility and adaptability enable them to infect plants, animals, and humans, resulting in a rising number of Fusarium-related diseases worldwide. Over the past two decades, there have been more reports of fusariosis in humans, especially in immunocompromised populations [20]. Similar to this, significant outbreaks of Fusarium head blight have recently (in 2022) led to epidemic-level disease incidence in Ethiopia, resulting in significant yield losses in wheat-growing regions [21]. This multifaceted menace brings a growing concern for plant pathologists, clinicians, veterinarians, and food safety experts. Therefore, acquiring a comprehensive understanding of *Fusarium* pathogenic mechanisms, environmental resilience, and host–pathogen interactions is critical for developing integrated strategies to mitigate its impact across sectors.

Despite growing recognition of *Fusarium* as a cross-kingdom pathogen that causes deleterious impacts on agriculture and human health, current knowledge remains scattered across disciplines, with plant pathology, veterinary science, and medical mycology often addressing these threats separately. This study summarizes the existing knowledge on *Fusarium* as a cross-kingdom pathogen, emphasizing its taxonomy, virulence mechanisms, toxin production, and novel challenges in the detection and management of this versatile pathogen. An interconnected approach to counter this globally pervasive threat is highlighted, in which human, animal, and plant health are unified under the “One Health” concept.

## 2. *Fusarium* Infection

### 2.1. Fusarium Infection in Plants

*Fusarium* species are considered as one of the most devastating plant pathogens due to their ability to produce mycotoxins [22]. They infect a wide range of plant hosts, which are vital for human and animal nutrition including major staple, horticultural and cash crops such as cereals (wheat, maize, and rice), banana, legumes, and vegetables [4,23,24]. They target various crop parts such as grains, seedlings, roots, heads, or stems, leading to a range of disease manifestations, reducing commercial yield, and a decline in product quality [25]. *Fusarium* infections lead to wilting, root rot, and vascular dysfunction, often resulting in significant yield losses as well as crop failure. For example, Panama disease is a classic example of the devastating impacts of *Fusarium,* which has caused catastrophic losses in global banana production, leading to a dramatic industry-wide transition to Cavendish varieties [24]. Due to the persistence of pathogens in soil, affected plantations frequently experience vast yield losses and long-term land abandonment. In addition to causing billions of dollars in economic losses, the disease is a serious threat to food security and global banana production [24]. Fusarium Head Blight (FHB), on the other hand, is a major disease of grains such as wheat and barley, particularly in temperate regions. Wheat Head Blight (WHB), caused by *F. graminearum,* is a major epidemic disease of wheat that directly affects global food security. It has the potential to cause yield losses ranging from 10% to 70%, impacting over 70,000 hectares of farmland [26]. Even though the yield losses are moderate, the economic loss caused by this is amplified by strict food and safety regulations, making it one of the costliest among all the *Fusarium*-associated diseases [27].

#### Mechanism of Infection in Plants

*Fusarium* infection in plants typically begins with host entry through natural openings, wounds, or root zones, where the fungus establishes initial contact with plant tissues. In plants, *Fusarium* primarily enters through natural openings. Most *Fusarium* species typically invade the husks of wheat and barley through pre-existing natural openings, such as stomata [28]. Cracks caused by emerging lateral roots, through the root cap, root hairs or branch roots or wounds also facilitate the penetration of these fungi [29,30,31,32]. For instance, when *F. oxysporum* infects banana, the pathogen enters through the epidermis and advances through intercellular spaces to reach the vascular bundles [33]. In tomato, entry occurs through the elongation zone or the root tip in hydroponic culture and through the root epidermal cells of the collar region in soil culture. Following successful penetration, *Fusarium* species colonize host tissues and spread systemically, most notably through the vascular system. Moreover, *F. oxysporum* infects plants by penetrating the root system, then moving through the epidermis, cortex, and endodermis to reach the xylem vessels. Then it spreads upwards by sporulating and using the xylem stream to carry microconidia. Conidia germinate at vessel ends, facilitating the colonization [34]. The nature of progression towards the vascular bundle through intercellular and intracellular spaces depends on the host [35]. The time course of the infection process and its extension into vascular tissues vary with the host, pathogen race, and cultivar. For instance, when comparing two races of banana infected by *F.oxysporum* f. sp. *cubense* (Foc), Foc 1 & Foc 4, the latter shows greater aggressiveness and invasive capacity than Foc 1 [33]. When wilt-susceptible cultivar (JG62) was compared with a wilt-resistant cultivar (Digvijay) against Foc race 2, notable differences in the penetration pathways were observed. Even though Foc 2 entered the resistant cultivar, its proliferation was majorly restricted [36].

To facilitate tissue invasion and nutrient acquisition, *Fusarium* deploys a suite of cell wall–degrading enzymes that dismantle structural barriers of the host. During infection, the fungus breaches the intricate physical defense structures within the cell walls of the host plant [37,38]. After penetrating the above-mentioned pathways, they secrete a variety of cell wall-degrading enzymes (CWDE) to further penetrate into the cells [29]. Genomic studies of plant-pathogenic *Fusarium* have identified numerous enzymes capable of degrading plant cell walls, thereby facilitating direct entry into host tissues [35]. Mostly this is a combination of enzymes, including cellulases, cutinases, pectinases, proteases and xylanases [39,40]. These released hydrolytic enzymes that degrade cell wall polymers, enable *Fusarium* to infiltrate plant tissues and absorb the nutrients released in the process [39]. They derive nutrients from cytoplasmic fats, proteins and starch, which become accessible only after being broken down by the enzymes they secrete [39].

In addition to polysaccharide-degrading enzymes, proteases play a critical role in *Fusarium* pathogenicity by targeting host proteins involved in defense and cellular integrity. In addition to CWDE, *Fusarium* actively produces proteolytic enzymes during plant infection to penetrate tissues, evade host defenses, and utilize host nutrients. FolAsp, a secreted aspartic protease by *F. oxysporum* f. sp. *lycopersici (*Fol), the causative agent of tomato wilt, degrades host proteins, facilitating deep penetration, and its overexpression enhances the fungal virulence [26]. Similarly, FoAPY1, a peptidase enzyme secreted by *F. oxysporum,* enhances the tomato pathogen virulence by degrading host defense-related proteins [41].

Beyond physical tissue degradation, *Fusarium* actively suppresses host immune responses by producing mycotoxins and other secondary metabolites. Upon identifying a threat due to a pathogen, plants develop innate immunity responses to combat the infection process. They secrete toxins and activate defense signaling pathways, including reactive oxygen species (ROS) production, lignification, and pathogenesis-related proteins [35,42]. However, *Fusarium* pathogens can challenge these responses by secreting mycotoxins. For example, *F. oxysporum* suppresses these immune responses by secreting Fusaric acid. This mycotoxin interferes with the antioxidant enzymes such as catalase and peroxidase, thereby reducing ROS accumulation. This suppression weakens the innate immune mechanisms of the plant and increases infection severity [43].

Under specific environmental or host conditions, some *Fusarium* species can also form specialized infection structures that enhance host penetration. In typical scenarios, *Fusarium* does not naturally produce specialized penetration structures such as appressoria or haustoria; rather, it relies on natural openings or direct penetration via short infection [39]. However, contrasting findings have been reported, indicating that under certain conditions, *Fusarium* may exhibit the formation of specialized structures that facilitate penetration [28,44]. According to those studies, during a *Fusarium* head blight (FHB) infection, *F. graminearum* has the potential to form lobate appressoria and infection cushions [28]. Compound appressoria formed on various tissue types, including caryopses and husks, indicating that appressorium formation occurs independently of tissue type.

### 2.2. Fusarium Infections in Humans and Animals

*Fusarium* is responsible for a wide range of infections in humans, including superficial, locally invasive, and systemic infections. Among the diseases caused by this genus, the most prominent are Fusarium Keratitis, Cutaneous Fusariosis, and Onychomycosis. Approximately ten *Fusarium* species have been identified to be associated with human pathogenicity, including *F. chlamydosporum*, *F. concolor*, *F. dimerum*, *F. fujikuroi*, *F. incarnatum-equiseti*, *F. lateritium*, *F. oxysporum*, *F. sambucinum* and *F. solani* (*Neocosmospora solani*) [6,45]. Within these species complexes, *F. solani* (*Neocosmospora solani*) is the most frequently encountered and aggressive, accounting for roughly 40–60% of reported infections. This is followed by *F. oxysporum,* responsible for about 20%, and *F. fujikuroi,* along with *F. moniliforme,* which together contribute approximately 10% [20]. The manifestation of fusariosis is primarily influenced by the host’s immune status and the route of pathogen entry [46,47].

In addition to human disease, *Fusarium* species are also capable of causing infections in a range of animal hosts, although such cases are comparatively rare. Several *Fusarium* species have been identified as causing infections in animals, each associated with a particular host and disease manifestations. The most significant impact of *Fusarium* in veterinary sciences arises from the various mycotoxicoses induced in animals contaminated with *Fusarium* toxins, including estrogenic compounds, fumonisins and trichothecenes [48]. However, according to the available literature, veterinary fusarioses are rare, and reported cases are typically associated with economically valuable domestic animals. Among infections caused by *Fusarium* sp. in animals, keratitis and dermatitis are the most common and frequently observed conditions [49]. Rare *Fusarium* infections include melanized lesions on the abdominal segments and cephalothorax of crustaceans, dysecdysis in snakes, embryonic mortality in sea turtle eggs, and invasive sinusitis with facial mycetoma in dogs. As further explained by the same author, *F. solani* (*Neocosmospora solani*) is the primary species associated with veterinary strains [48].

#### Mechanism of Infections in Humans and Animals

In humans and animals, *Fusarium* infections typically occur through wounds, inhalation, or ingestion, with disease severity strongly influenced by host immune status*. Fusarium* primarily causes diseases in immunosuppressed individuals, where infections can be severe or invasive, while cases in immunocompetent hosts are rare and typically mild or superficial [46,50]. The pathogenesis of the invasive *Fusarium* spp. is associated with the disruption of the mucosa or cutaneous barrier of the host cell, and the fungi enter through minor skin or mucous membrane breaks, often from soil or water, leading to localized infections. In immunosuppressed individuals, these sites become entry points for systemic dissemination. Infections can also result from extensive skin damage, such as burns and wounds, airborne conidia or the presence of foreign bodies, including contact lenses (keratitis) and peritoneal dialysis catheter [51].

Following entry into the host, the infection progresses through successive stages. When inhaled, conidia germinate into hyphae in the alveoli, leading to inflammation and bronchial dissemination [52]. Subsequently, hyphae invade blood vessels, causing thrombosis and tissue infarction. Furthermore, once they penetrate animal tissues, *Fusarium* produces small yeast-like structures called aleuroconidia that invade blood vessels, causing fungemia and dissemination to various organs, including the skin [53]. *Fusarium* exhibits organ-specific invasion strategies [54]. In their mouse model study, hyphal growth was observed in the kidney and heart. At the same time, in the spleen, liver, and lungs, the fungus formed chlamydospore-like structures, thick-walled cells associated with stress tolerance and long-term persistence. Moreover, the formation of biofilm is another strategy that *Fusarium* has employed. Biofilm formation has been demonstrated to be particularly associated with keratitis and onychomycosis in corneal infections, contact lens infections, and nail infections, emphasizing long-term persistence and therapeutic challenges [55].

Similar to plants, proteolytic enzymes play a crucial role in *Fusarium* pathogenesis by facilitating tissue invasion, immune evasion, and nutrient acquisition within the host. *Fusarium* species are recognized for secreting various types and quantities of extracellular proteases, which enhance virulence [56].

### 2.3. Similarities and Dissimilarities Between the Infection Mechanisms of Plants and Animals

*Fusarium* infections in plants and animals exhibit both similarities and differences in their infection processes and in the impacts on the host. The primary route of infection in plants is through intercellular spaces toward the vascular system after entry via roots, wounds, and natural openings. The process relies heavily on cell wall-degrading enzymes and phytotoxic metabolites, which facilitate vascular blockage and suppress plant immune responses. In contrast, infections in humans and animals typically occur via wounds, inhalation, or mucosal disruption, particularly in immunocompromised hosts, and dissemination occurs via the bloodstream rather than through specialized vascular tissues. Virulence in animal hosts relies less on structural tissue degradation and more on survival-oriented mechanisms such as stress tolerance, biofilm formation, vascular invasion via aleuroconidia, and persistence under immune pressure.

**Figure 1:**
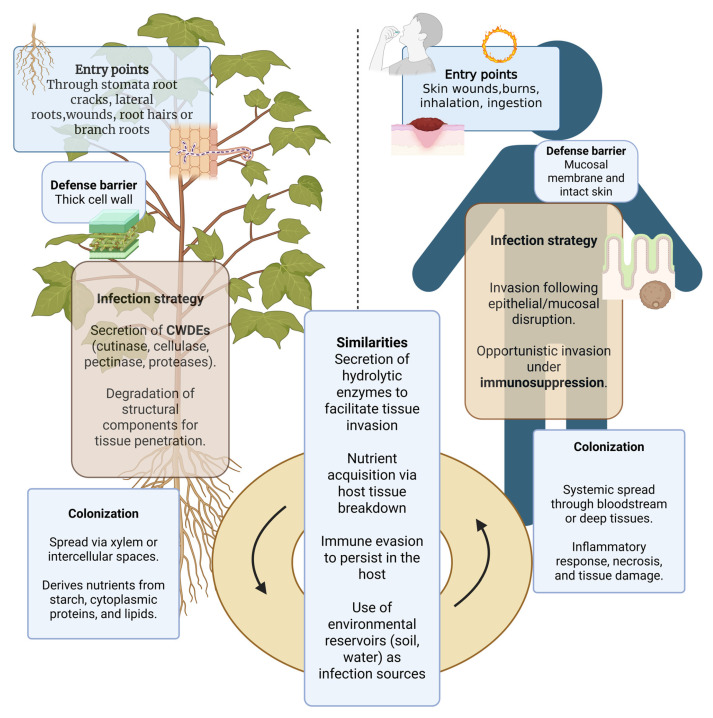
Similarities and dissimilarities in *Fusarium* infection in animals and plants.

During infection, both plant and animal pathogens deploy a range of proteolytic enzymes to invade the host. This functional similarity in proteolytic-mediated virulence strategies across kingdoms suggests that *Fusarium* can adapt and infect both plants and animals. In addition to that, *F. oxysporum* secretes Fusaric acid, a mycotoxin known to suppress host immune responses in both kingdoms [35]. These conserved infection strategies demonstrate their ability to exploit shared host weaknesses, enabling successful adaptation and infection in both kingdoms (Figure 1).

Beyond mechanistic similarities and differences, shared environmental reservoirs play a critical role in facilitating cross-kingdom exposure to *Fusarium* species. In addition to their shared infection mechanisms, environmental exposure plays a critical role in facilitating cross-kingdom pathways, particularly for opportunistic pathogens such as *Fusarium.* These fungi are commonly found in soil, water, decaying vegetation, and plant surfaces where they get the opportunity to interact with a wide range of hosts [50]. Even though this genus is commonly classified as soil-dwelling due to its abundance in soil, it is also found in aquatic environments, including various water-related sources such as public swimming pools, shower drains, and hospital water systems [57]. Natural ecosystems serve as reservoirs for their genetic diversity, encompassing both newly identified species and those already known or with the potential to act as pathogens [58]. For instance, agricultural workers and individuals in close contact with soil or plants may be at higher risk of developing infections. In the same vein, aquatic environments contaminated with *Fusarium* can serve as reservoirs that contribute to infections in aquatic animals and, possibly, humans through indirect routes. These diverse environmental interfaces not only support the survival and spread of *Fusarium* but also promote its genetic adaptability, enhancing its ability to infect across kingdoms.

## 3. Host Adaptability and Cross-Kingdom Pathogenesis of *Fusarium*

As a highly diverse fungal genus comprising several hundred species, some plant pathogenic species infect only a single host, and others exhibit a broad host range affecting multiple plant species [59]. Despite the significant differences in within-plant and animal host tissues, nearly all 24 *Fusarium* taxa identified in human infections have also been associated with plant diseases [59,60]. Interestingly *Fusarium* species isolated from keratamycosis cases retain the ability to infect plants and subsequently re-colonize other human tissues, such as nails, providing key evidence of cross-kingdom pathogenicity [61]. Notably, *F. musae* has been isolated from both banana fruits and immunocompromised patients [15]. Remarkably, isolates from human infections often shared the same genetic profiles as those found in a variety of environmental sources, including hospitals [16]. This suggests that human infections are likely the result of incidental exposure of vulnerable patients to *Fusarium* strains present in their environment. As further explained by similar authors, *Neocosmospora* species complex-associated mycoses in humans and other animals originate from a broad phylogenetic spectrum, highlighting the extensive adaptability and infection potential within this diverse genus of fungi. Together, these observations suggest that host switching in *Fusarium* is not accidental but reflects a shared genomic capacity for cross-kingdom adaptation, underpinned by broad environmental versatility.

## 4. Environmental and Evolutionary Factors Affecting Cross-Kingdom Pathogenesis of *Fusarium*

Ability of *Fusarium* species in transcending host kingdom boundaries results from both environmental selection and evolutionary innovation acting on their genomes. In a 2024 study, comparative genomic analyses were performed, and the genomes of *F. keratoplasticum* and *F. petroliphilum*, two members of the FSSC known to cause human keratitis, were compared with those of other *Fusarium* species with plant/animal pathogen capacity [62]. Results revealed that human pathogenicity evolved independently across multiple lineages, and, as shown by pan-genome and transcriptomic analyses, there is high genetic variability, including accessory chromosomes and conserved secondary-metabolite gene clusters potentially linked to infection [62]. Additionally, transcriptomic analysis under stress conditions indicated that these species are highly adapted to environments relevant to human infection, emphasizing their growing potential as cross-kingdom pathogens in the face of climate change [62]. To understand how *Fusarium* repeatedly achieves host switching, the following sections examine the genomic and environmental drivers that underpin cross-kingdom adaptation.

### 4.1. Comparative Genomic and Proteomic Insights into Cross-Kingdom Pathogenesis

#### 4.1.1. Core and Accessory Genome Architecture in *Fusarium*

The significance of differentiating between core and accessory chromosomes (or lineage-specific chromosomes) is highlighted by comparative genomic analyses. Accessory chromosomes disproportionately contribute to the wide genomic diversity seen in *Fusarium* pathogens, whereas core chromosomes encode crucial housekeeping genes necessary for fundamental cellular functions [63]. The existence of accessory chromosomes in plant-pathogenic *F. oxysporum* strains, emphasizing their crucial role in host-specific pathogenicity was first reported in 2010 [64]. That particular study solidified the presence of an accessory chromosome (AC) by examining the genomes of phenotypically diverse species: *F. graminearum*, *F. oxysporum* f. sp. lycopersici (Fol4287) and *F. verticillioides*. Among the pathogenicity-related genes located on lineage-specific (LS) chromosomes, well-known effector proteins, peptides that cause necrosis and ethylene, and a wide range of secreted enzymes that are thought to break down or alter plant and fungal cell walls to facilitate host colonization were identified. Furthermore, an expansion of genes involved in lipid metabolism and lipid-derived secondary messenger pathways is observed in AC regions, indicating a significant role for lipid signaling in fungal pathogenicity. The lineage-specific (LS) chromosome content varies significantly among formae speciales with different host specificities, consistent with the species-complex nature of *F. oxysporum.* Notably, LS chromosomes encode a number of small proteins secreted during colonization of the plant vascular system, such as the in planta-secreted oxidoreductase ORX1 and the virulence-associated effectors Six1 (Avr3) and Six3 (Avr2). Subsequent studies confirmed that AC-mediated host specificity is a common feature among plant-pathogenic *F. oxysporum* formae speciales, including strains infecting melon, onion, cucumber, and legumes [65,66]. For instance, two recent studies recorded the revelation of an entire AC that has been linked with host-specific pathogenicity in *F. oxysporum* f. sp. *lycopercisi* and *F. oxysporum* f sp. *radiciscucumerinum* [64,67].

After ten years from the initial report of the AC in phytopathogenic *Fusarium* strain, presence of AC in the two human-pathogenic *F. oxysporum* strains was reported, shedding light on the cross-kingdom pathogenicity of these versatile fungi [68]. The two human-pathogenic *F. oxysporum* isolates, NRRL 32931 and NRRL 47514, differed significantly from their plant-pathogenic counterparts in their lineage-specific (LS) chromosome composition and functional content, as determined by a comparative genomic analysis. A unique set of four LS chromosomes, distinct from those previously reported in phytopathogenic strains, was present in NRRL 32931, isolated from the blood of a leukemia patient with invasive fusariosis. Although different from plant-pathogenic isolates, NRRL 47514, recovered from a contact lens-associated keratitis outbreak, shared significant LS sequences with NRRL 32931, including genes related to metal-ion and cation transport. By allowing the pathogen to overcome host-imposed nutritional immunity and establish infection, these genetic traits are likely to facilitate adaptation to the mammalian host. Both human isolates lacked the distinctive signature effector motifs frequently linked to plant infection, in contrast to the genomes of phytopathogenic *F. oxysporum* species complex (FOSC) [69]. Furthermore, neither of the human-pathogenic genomes contained transposable elements associated with pathogenicity in plant-infecting strains, such as Helitrons and miniature impala (MIMP) elements. Rather, a unique repertoire of transposons, primarily AT-rich repetitive elements, was identified in these isolates, indicating distinct genome-remodeling mechanisms underlying host specialization in human-associated strains. Additionally, these human-infecting genomes lacked key plant-associated virulence factors, such as SIX (Secreted In Xylem) effector genes and enzymes involved in plant cell wall degradation that are common in phytopathogenic *F. oxysporum* strains [67,70,71]. AC in human clinical isolates were enriched with genes involved in metal ion and inorganic cations transport, whereas plant isolates carried genes encoding for secreted effectors and enzymes that degrade plant cell walls [72]. Many virulence-associated effectors are encoded on accessory chromosomes, and secreted effectors play a major role in *Fusariu*m plant infection by facilitating host recognition, immune suppression, and tissue colonization [64,73,74]. The importance of effector diversity in host specialization is highlighted by the fact that plant-pathogenic strains have larger repertoires of SIX effectors and carbohydrate-active enzymes necessary for complete virulence, whereas endophytic and human-pathogenic strains have fewer such effectors [67,71,75,76].

**Figure 2:**
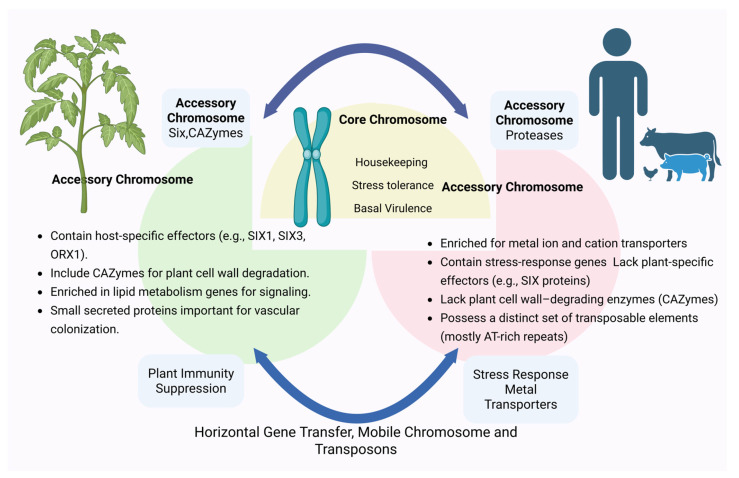
A conceptual model of genome compartmentalization in *Fusarium* and its contribution to cross-kingdom pathogenicity.

This introduces a new perspective on the role of AC in human pathogenicity and underscores the diversity and distinct characteristics of ACs in relation to cross-kingdom host adaptation (Figure 2).

#### 4.1.2. Mobile Chromosomes and Horizontal Gene Transfer as Drivers of Host Adaptation

Beyond differences in accessory chromosome content, the mobility of these chromosomes and their horizontal transfer play a pivotal role in shaping host adaptation across divergent ecological niches. Mechanisms such as horizontal gene transfer (HGT) and mutations have led to the emergence of highly virulent strains with enhanced pathogenicity and mycotoxin production. HGT is a crucial evolutionary strategy that enables fungi to acquire novel genetic material [77]. AC region harbors more than 74% of the transposable elements in the Fol genome, approximately 95% of all DNA transposons, elucidating their role in host pathogenic specificity. By enabling the acquisition of new characteristics that improve pathogenicity and environmental resilience, HGT has become a key mechanism driving the evolutionary adaptability of *Fusarium* species [64]. As documented in several studies, *Fusarium* species have incorporated specific genes, including those involved in biosynthesis through HGT, highlighting their evolutionary adaptability and genomic plasticity [77,78,79]. Lineage-specific chromosomes of *F*. *oxysporum* that are rich in transposons and pathogenicity-related genes can be horizontally transferred between strains, transforming nonpathogenic strains into virulent pathogens and illustrating the role of chromosome-level HGT in shaping host specificity and the emergence of new pathogenic lineages [64]. A genome-wide analysis of *F. verticillioides*, identified 36 genes likely acquired from bacteria via horizontal gene transfer, suggesting that HGT has contributed to the expansion of metabolic functions and enhanced adaptability of the fungus under various environmental stresses [80]. These results collectively highlight the importance of both intra- and inter-kingdom HGT events in the ecological success, diversity, and cross-kingdom infectiousness of *Fusarium* species, which make them harmful pathogens in plant and animal systems.

#### 4.1.3. Proteomic and Transcriptomic Signatures of Shared Virulence Mechanisms

By identifying conserved virulence factors and regulatory pathways expressed during infection, proteomic and transcriptomic analyses offer crucial insights into the molecular basis of pathogenicity. Similar mechanisms that allow pathogens to adapt to both plant and animal environments are highlighted by comparative profiling across hosts. Protein kinases are key regulators of virulence in *Fusarium*, coordinating host sensing, signal transduction, and adaptive responses to environmental cues, as revealed by proteomic and transcriptomic studies. A conserved core of kinase families shared by ascomycetes and an expanded repertoire of kinase genes within the species complex, partially driven by accessory chromosomes, are revealed by comparative kinome analyses of *F. oxysporum* [81]. Mpk1, Hog1, and Fmk1 contribute to colonization, virulence, and host penetration, and conserved MAPK (mitogen-activated protein kinase) pathways are important for stress adaptation, morphogenesis, and host interaction [82,83]. Furthermore, the growth of TOR (Target of rapamycin) and histidine kinases underscores the importance of environmental signal perception and nutrient sensing for this pathogen’s ability to adapt to a variety of plant and animal hosts [81,84]. The observed kinome expansions among members of the FOSC appear to have equipped the pathogens to survive in diverse hosts.

*Fusarium* functional division into a dynamic accessory genome made up of chromosomes unique to each lineage and a conserved core genome that codes for vital housekeeping functions plays a pivotal role in cross-kingdom pathogenicity adaptation. The accessory chromosomes are rich in genes linked to pathogenicity, such as effectors, carbohydrate-active enzymes, stress-response factors, and metal ion transporters, which together determine host-specific interactions, whereas the core chromosomes show high synteny and evolutionary stability. Because different gene sets can be acquired or lost without impairing essential cellular processes, the association of these virulence determinants with accessory chromosomes provides a mechanistic basis for cross-kingdom pathogenicity. Crucially, host switching and adaptation to new ecological niches are facilitated by the rapid acquisition of novel virulence traits, enabled by the mobility of accessory chromosomes and horizontal transfer. The enrichment of transposable elements within accessory regions, which encourage genome rearrangements, gene diversification, and regulatory innovation, further supports this genomic plasticity. An integrated evolutionary framework that facilitates both host specialization and the sporadic emergence of cross-kingdom pathogenicity in *Fusarium* is formed by mobile chromosomes, transposons, and horizontally transferable accessory gene clusters. Experimental evidence that a human-pathogenic strain can colonize plant roots and a plant-pathogenic strain can infect corneal tissue further supports this genomic plasticity, showing that shared and mobile genomic features enable cross-kingdom adaptation even when disease symptoms remain relatively mild [63].

### 4.2. Genetic Mutations and Race Evolution as Drivers of Host Adaptation

The differentiation of *F. oxysporum* into formae speciales and races illustrates the important role of genetic mutations in its evolutionary success. Genetic mutations in pathogenicity-associated genes, such as deletions and point mutations in effector genes like AVR1 and AVR2, have played a critical role in the evolutionary adaptation of *F. oxysporum* [85,86,87,88]. These mutations lead to the emergence of new pathogenic races capable of overcoming host plant resistance, thereby allowing pathogens to persist and spread across different cultivars [89]. Additionally, the complex relationships between vegetative compatibility groups (VCGs) and races highlight the genetic diversity within formae speciales, enabling rapid adaptation to changing environmental pressures and host defenses. Furthermore, single-nucleotide polymorphisms in *Fusarium* contribute to genetic variation in secreted in xylem (SIX) gene sequences, supporting adaptation to changing environments and host defenses [90,91,92]. Specific mutations in the AVR2 gene allow *F. oxysporum* strains to overcome 1–2-mediated resistance by altering amino acids, leading to loss of avirulence without compromising virulence [86,88,93]. Similar mutations are likely in the AVR1 and AVR3 genes, suggesting a broader mechanism for resistance breakdown [87]. Together, these mutations and the dynamic reshuffling of genetic material have strengthened the ability of this pathogen to evolve and expand its host range, and maintain its pathogenic success over time.

### 4.3. Environmental Factors

Shifts in climate conditions are expected to significantly influence the distribution and survival of *Fusarium* species. Such climatic changes can create new, suitable habitats that were previously unsuitable, thereby enabling the expansion of ecological niche of *Fusarium*. As a result, these fungi can colonize larger geographic regions and pose a greater threat of infection to plants and animals across different ecosystems [94,95].

Atmospheric conditions, particularly temperature and moisture levels, play a fundamental role in shaping fungi. Among these, temperature is a crucial environmental factor that influences pathogenesis at multiple stages of the fungal life cycle, from spore germination and hyphal growth to reproduction and infection. Even the slightest fluctuations in temperature can significantly affect the reproduction rates of fungal pathogens, alter the frequency and success of infection cycles, and impact both the short-term spread and long-term dispersal potential of fungal spores [96]. In addition to temperature, other environmental factors such as humidity, soil composition, and agricultural practices collectively influence the survival, transmission, and overall virulence of *Fusarium* species. However, temperature exerts a particularly pronounced effect on the fungus, governing its growth dynamics, promoting sporulation, and affecting the vulnerability of the host plants to infection [2,97]. As global temperatures continue to rise, fungal populations, including *Fusarium* species, are predicted to undergo stress-induced adaptations that enhance their heat tolerance. These evolutionary adjustments may enable *Fusarium* to thrive under high temperatures and could potentially contribute to increased pathogenicity not only in plants but also in human hosts [98]. Furthermore, other atmospheric variables such as humidity, wind patterns, and light intensity are equally significant in influencing fungal behavior. They affect the production, maturation, and dispersal efficiency of asexual reproductive structures, including conidia and ascospores, thereby playing a crucial role in the epidemiology and seasonal dynamics of *Fusarium* infections [99,100].

In addition, extreme rainfall events, another consequence of a changing climate, also play a pronounced role in enhancing the spread and the severity of *Fusarium* infections by creating favorable conditions. For instance, heavy rainfall can lead to an increase in the number of *Fusarium* wilt cases, highlighting how it facilitates the spread and severity of these microbes [101]. Moreover, *Fusarium* species have been detected in rainwater samples, suggesting that rainfall can act as a vector for dispersing these pathogens [102].

Climate change has direct, adverse effects, not only by enhancing pathogenic *Fusarium* reproduction but also by altering or increasing mycotoxin production within the genus, thereby affecting food safety. Since mycotoxin-producing species favor distinct climatic environments, the occurrence and types of mycotoxins vary widely across geographic regions [103]. Climate change will alter the geographic distribution of mycotoxin-producing fungi, leading to changes in global mycotoxin occurrence patterns. With rising global temperatures, an overall increase in mycotoxin-contaminated crops is anticipated; however, regional differences may result in both increases and decreases in contamination levels [104,105,106,107].

The success of *Fusarium* as a cross-kingdom pathogen is attributable to its genomic plasticity, conserved virulence traits, and extensive environmental exposure. The presence of the flexible AC with mobile genetic elements enables *Fusarium* to adapt to a diverse range of hosts. Moreover, genetic mutations and evolutionary adaptation ensure host adaptation, while shared virulence mechanisms support infection across fundamentally different biological systems. Attributing to its environmental ubiquity, *Fusarium* can act as an opportunistic pathogen exploiting susceptible plant, animal, or human hosts when conditions permit.

## 5. Novel Methods to Study Cross-Kingdom Infection

Within a “One Health framework”, understanding cross-kingdom pathogenicity in *Fusarium* requires integrative approaches that link plant, animal, and human systems through shared data, methods, and surveillance strategies. With an increasing number of *Fusarium* cases associated with animals, identifying and exploring novel methods to study cross-kingdom pathogenicity in this versatile pathogen is vital. According to the literature, tools such as comparative genomics, host transcriptomics, model organisms (e.g., *Galleria mellonella*), and CRISPR-based functional validation have shown promising impacts.

### 5.1. Comparative Genomics

Comparative genomics has emerged as a powerful approach to elucidating the molecular mechanisms underlying cross-kingdom pathogenicity in *Fusarium*. Recent developments in sequencing technologies and genome assembly algorithms have made it possible to perform high-resolution analyses of conserved and lineage-specific genomic regions linked to virulence, in addition to defining evolutionary relationships [108,109]. Across biological kingdoms, this method has been extensively used to identify conserved virulence determinants and elucidate host–pathogen interactions. For example, comparative genomic and transcriptomic analyses have been used to investigate the cross-kingdom pathogenicity of pathogens in the *F. solani* species complex (FSSC) [14]. Results revealed that FSSC, usually known for infecting plants, can also infect animals using similar infection strategies, such as penetration, colonization, and tissue degradation, on turtle eggs as it does on plant hosts. Importantly, genes usually linked to plant pathogenesis (such as cellulase and CEFM-domain proteins) were also activated during animal infections, suggesting that these fungi might use a common genetic toolkit across kingdoms. Furthermore, especially in agricultural settings where human, animal and plant interactions are intensified, integrative genomic approaches make it easier to identify accessory genomic regions and adaptive traits that may encourage host shifts and opportunistic infections. These multi-omics approaches go beyond descriptive analyses by combining pathogen genomics and host transcriptomic responses. Instead, they directly unravel the genetic mechanisms that facilitate spillover events and the emergence of cross-kingdom diseases, while also providing guidance for the development of diagnostic tools and disease surveillance initiatives [108].

### 5.2. Host Transcriptomics

During fungal infection, *Fusarium* pathogens can induce distinct gene-expression patterns in host cells. Gaining detailed insights into this phenomenon can enhance understanding of the infection process, enabling effective treatment and control to combat cross-kingdom-associated pathogenicity [110]. While efforts to understand the molecular mechanisms of fungal pathogens and host cells remain in their infancy, high-throughput transcriptomic analysis using next-generation sequencing (RNA-seq) is a promising approach for understanding pathogen-triggered host responses [110]. Host transcriptomics, defined as qualitative or quantitative studies of RNAs at a genome-wide scale, is a valuable method for uncovering both diagnostic markers and the underlying immune mechanisms involved in infectious diseases [111,112]. Here, the gene expression of the host is studied in response to an infection. Mapping host transcriptional changes can aid in identifying disease biomarkers and genes associated with resistance or susceptibility to cross-kingdom pathogens [113]. Therefore, host transcriptomic analysis is a promising method for clarifying conserved and host-specific molecular responses during infection, providing important insights into the mechanisms underlying cross-kingdom pathogenicity, even though direct cross-kingdom transcriptomic studies in *Fusarium* remain scarce.

### 5.3. Model Organisms

Another promising approach recently used by scientists to investigate cross-kingdom pathogenicity is the use of diverse model organisms that simulate both plant and animal host environments. Several invertebrate host models have been developed, enabling multidisciplinary investigations of host-fungal interactions from both the host and pathogen viewpoints [114]. Mammalian models, especially mice, have played a crucial role in uncovering the virulence strategies of microbial pathogens [115]. However, attention has recently been drawn to non-invertebrate models due to the expense, complexity, and time-consuming nature of vertebrate models [116]. A study in 2011 demonstrated the potential of *Galleria mellonella* as a non-vertebrate model for investigating the virulence mechanism of *F. oxysporum* in animal hosts. The fungus demonstrated true invasive infection by multiplying within the larval hemocoel, killing the insect, and colonizing it. This suggests that *F. oxysporum* can infect phylogenetically diverse hosts through conserved virulence mechanisms [116]. These findings highlight the potential and efficiency of using model organisms to investigate virulence mechanisms and support its application in studies exploring the cross-kingdom pathogenicity of *Fusarium*.

### 5.4. CRISPR-Based Functional Validation

Another powerful advancement in studying gene function in relation to cross-kingdom pathogenicity is the use of CRISPR-based functional validation, which enables precise identification and manipulation of pathogen genes to confirm the roles of virulence genes. CRISPR-Cas9 offers a rapid, efficient, and cost-effective method for high-throughput screening of multiple genes simultaneously [117]. It can be used to test and confirm the roles of specific genes in various biological processes, such as virulence and host specificity [118]. In addition to functional genomic screens, CRISPR-Cas9 has been used in the past to create precise genetic models of disease [119,120], offering a powerful platform to study and address mechanisms underlying cross-kingdom pathogenicity. As CRISPR-based technology enables rapid generation of complex genetic models of disease in vivo, this application facilitates the examination of gene–environment interactions. This enables the development of diseases and drug resistance, leading to precision in targeted therapy design [117]. In the context of *Fusarium,* targeted disruption, base editing, and tagging of genes involved in virulence and metabolism are made possible by the successful development and optimization of CRISPR-based gene editing systems [121,122,123]. Thus, this can be utilized as a necessary experimental platform to validate candidate virulence determinants identified through comparative genomics and transcriptomic analyses. Even though CRISPR-based studies that directly investigate cross-kingdom pathogenicity are limited, the established genome-editing platforms within this genus present a substantial opportunity to functionally validate conserved virulence genes in both plant and animal hosts, as well as to clarify the genetic mechanisms that facilitate cross-kingdom infection.

## 6. Mycotoxins and the One Health Perspective

**Figure 3:**
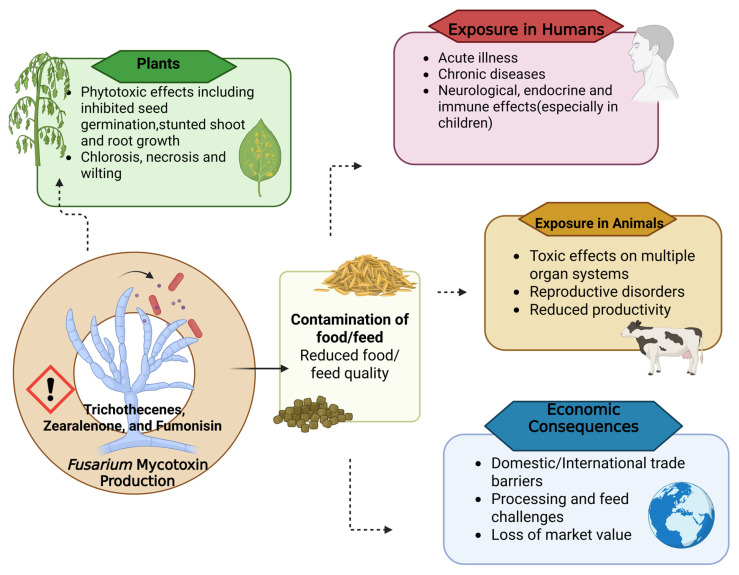
Pathways of mycotoxin impact of *Fusarium* from plants, human and animal health to global trade.

In addressing the harmful effects of *Fusarium* species, it is crucial to emphasize the significance of mycotoxins, which pose one of the most serious risks to human and animal health (Figure 3). Many species of *Fusarium* produce a range of secondary metabolites that elicit diverse physiological and pharmacological responses in plants and animals. It is among the most prevalent genera of plant-pathogenic fungi, and its mycotoxins have a greater overall impact than any other known toxin or toxin group [124]. They act as a way for the genus to reduce the number of superfluous precursors; this particular compound is not necessary for the growth and development of the fungi [125]. Although it confers only a minimal advantage over fungi, even low concentrations can have detrimental effects in humans and animals. Mycotoxin contamination in grains reduces grain quality for human consumption and creates challenges in processing and using the affected grain as animal feed [126]. It can cause acute or chronic illnesses, leading to death in some instances, while also adversely affecting animal productivity and disrupting both domestic and international trade [7,8,9]. In humans, children are particularly susceptible to the toxic effects of mycotoxins due to their greater sensitivity to immunological, neurological, endocrine, and neurotoxic effects, as well as their relatively lower body weight compared to adults [127]. Given the pervasive impact of mycotoxins across ecosystems, agricultural production, and human health, addressing these risks requires an integrated approach that recognizes the interconnectedness of environmental, animal, and human health, a core principle of the One Health framework.

**Figure 4:**
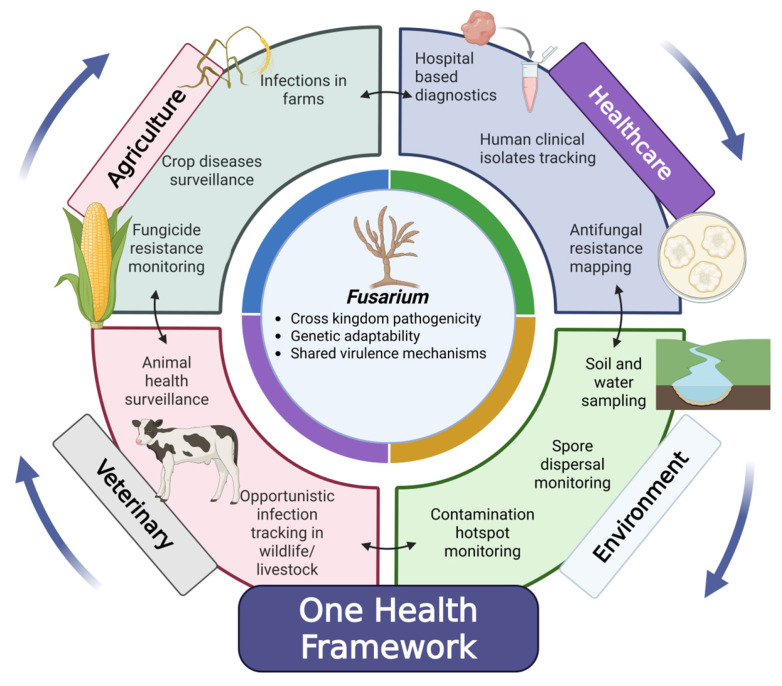
One health framework to build interconnectedness among related sectors.

In 2022, the World Health Organization listed the genus *Fusarium* among the high priority fungal pathogens requiring urgent research due to significant knowledge gaps. These include poor diagnostic tools, limited data on how infections spread, and natural resistance to antifungal treatments, factors that contribute to a high mortality rate of 43–67% [128]. Therefore, combating the challenges associated with cross-kingdom pathogenicity requires a unified approach. A combined diagnostic-surveillance framework that includes both agricultural and medical isolates is essential for thoroughly understanding and managing cross-kingdom pathogenicity. As suggested by genomic analysis, this versatile pathogen can infect both crops and humans/animals through conserved virulence mechanisms that enable it to cross various biological barriers. Traditional approaches that separately focus on either agricultural isolates or medical isolates are a major pitfall that has been associated with the exploration of interconnectedness. This results in missing important connections among disease outbreaks across different hosts. By unifying diagnostic data from farms, hospitals, and wildlife into a single surveillance system, researchers can examine emergence patterns, monitor genetic changes associated with virulence, adaptation, and resistance, and identify areas at high risk for spillover. This aligns with the principles of the “One Health approach,” which emphasizes the necessity of prioritizing the interconnectedness of human, animal, and plant health. Ultimately, such integration can support early detection and more accurate risk mapping of emerging infectious diseases with potential cross-kingdom cases (Figure 4).

## 7. Future Directions

The extensive combustion of fossil fuels and large-scale deforestation over the past few centuries have increased atmospheric greenhouse gas concentrations, which, in turn, have driven substantial climate change worldwide [129]. As climate plays a crucial role in regulating the growth, distribution, and severity of *Fusarium* sp. infections, factors such as temperature, humidity, and precipitation directly impact fungal sporulation, dispersal, and host susceptibility, ultimately influencing the prevalence and intensity of *Fusarium*-related diseases in both plants and humans [98,130,131,132]. According to a recent study, *Fusarium* spp. are able to adapt and expand their distribution in response to climate change, with significant shifts projected in Asia, Europe, Australia, and the Americas [133]. As environmental conditions favor its proliferation, *Fusarium* poses an increasing threat to crops, ecosystems, and potentially human and animal health, highlighting the urgent need for proactive mitigation strategies and future research.

Furthermore, crops experiencing environmental stresses, such as drought or episodes of excessive rainfall, tend to become more vulnerable to fungal attacks, thereby offering more opportunities for *Fusarium* infections to take hold [134,135]. As global warming continues, *Fusarium* is projected to invade regions previously unsuitable for its survival, thereby increasing its impact on natural ecosystems and agricultural productivity and potentially posing greater threats to animal and human health [98].

Moreover, the ecological overlap between plant-associated and clinical isolates makes it nearly impossible to distinguish pathogenic *Fusarium* isolates from opportunistic or environmental strains solely on the basis of morphological characteristics [136]. Host origin is inadequate to predict pathogenic potential or clinical risk [137]. In addition to this, the intrinsic resistance of *Fusarium* towards anti-fungal medications, for example, azoles and echinocandins, is also linked with the high mortality rate and limited treatment choices [138]. This intrinsic resistance, along with the environmental ubiquity of *Fusarium* and its impact on agriculture, highlights the role of this genus in a “One Health Framework” where plants, animal, and human health are interconnected.

Given the multitude of factors favoring *Fusarium*, proactive measures grounded in science and technology are essential to mitigate the impact of this versatile pathogen. To effectively address the associated challenges, it is essential to promote cross-sectoral collaboration among plant pathology, veterinary science, and human medicine. Implementing and promoting sustainable agricultural practices may help minimize the vulnerabilities faced by the agricultural sector. Strengthening regulations on mycotoxin contamination in food and feed is also essential to safeguard public and animal health. Incorporating climate change projections into disease forecasting models will enhance preparedness and may help minimize the adverse impacts. Furthermore, promoting education and awareness among key stakeholders, such as farmers, veterinarians, and public health officials, on cross-kingdom risks and prevention is essential for comprehensive, long-term management of the harmful impacts.

## 8. Conclusions

Although *Fusarium* is widely recognized as a plant pathogen, this review highlights its growing significance as a cross-kingdom threat that can infect plants, animals, and humans. This ability to cross host boundaries is driven by a combination of genomic plasticity, environmental pressures, and climate change, underscoring the relevance of *Fusarium* in a One Health context. Addressing this multifaceted threat will require integrated surveillance, advanced molecular and genomic tools, and coordinated collaboration across plant pathology, veterinary science, and human medicine.

## Data Availability

Data sharing is not applicable. No new data were created or analyzed in this study.

## Abbreviations

The following abbreviations are used in this manuscript:

AC	Accessory Chromosome
AVR	Avirulence
CC	Core Chromosome
LS	Lineage specific
HGT	Horizontal Gene Transfer
FOSC	*Fusarium Oxysporum* species complex
FSSC	*Fusarium Solani* species complex
SIX	Secreted in xylem
ROS	Reactive oxygen species
MAPK	Mitogen-Activated Protein Kinase
Hog1	High Osmolarity Glycerol response 1
MIMP	Miniature impala
CEFM	Common in Fungal Extracellular Membrane
VCG	Vegetative compatibility group
TOR	Target of rapamycin
